# Imaging and Characterization of Sustained Gadolinium Nanoparticle Release from Next Generation Radiotherapy Biomaterial

**DOI:** 10.3390/nano10112249

**Published:** 2020-11-13

**Authors:** Romy Mueller, Michele Moreau, Sayeda Yasmin-Karim, Andrea Protti, Olivier Tillement, Ross Berbeco, Jürgen Hesser, Wilfred Ngwa

**Affiliations:** 1 Department Data Analysis and Modeling in Medicine, Mannheim Institute for Intelligent Systems in Medicine (MIISM), Heidelberg University, 69117 Heidelberg, Germany; juergen.hesser@medma.uni-heidelberg.de; 2 Department of Radiation Oncology, Brigham and Women’s Hospital, Dana-Farber Cancer Institute, Boston, MA 02115, USA; Michele_Moreau@dfci.harvard.edu (M.M.); syasmin-karim@bwh.harvard.edu (S.Y.-K.); Ross_Berbeco@dfci.harvard.edu (R.B.); wngwa@bwh.harvard.edu (W.N.); 3 Department of Radiation Oncology, Harvard Medical School, Boston, MA 02115, USA; 4 Department of Physics, University of Massachusetts Lowell, Lowell, MA 01854, USA; 5 Department of Imaging, Lurie Family Imaging Center, Center for Biomedical Imaging in Oncology, Dana-Farber Cancer Institute, Harvard Medical School, Boston, MA 02110, USA; Andrea_Protti@dfci.harvard.edu; 6 Institut Lumière Matière, CNRS, Université de Lyon, 69622 Villeurbanne, France; olivier.tillement@univ-lyon1.fr; 7 Interdisciplinary Center for Scientific Computing (IWR), Heidelberg University, 69120 Heidelberg, Germany; 8 Central Institute for Computer Engineering (ZITI), Heidelberg University, 68159 Mannheim, Germany

**Keywords:** gadolinium-based nanoparticles, magnetic resonance imaging, biomaterials, quantitative magnetic resonance imaging

## Abstract

Smart radiotherapy biomaterials (SRBs) present a new opportunity to enhance image-guided radiotherapy while replacing routinely used inert radiotherapy biomaterials like fiducials. In this study the potential of SRBs loaded with gadolinium-based nanoparticles (GdNPs) is investigated for magnetic resonance imaging (MRI) contrast. GdNP release from SRB is quantified and modelled for accurate prediction. SRBs were manufactured similar to fiducials, with a cylindrical shell consisting of poly(lactic-co-glycolic) acid (PLGA) and a core loaded with GdNPs. Magnetic resonance imaging (MRI) contrast was investigated at 7T in vitro (in agar) and in vivo in subcutaneous tumors grown with the LLC1 lung cancer cell line in C57/BL6 mice. GdNPs were quantified in-phantom and in tumor and their release was modelled by the Weibull distribution. Gd concentration was linearly fitted to the R_1_ relaxation rate with a detection limit of 0.004 mmol/L and high confidence level (R^2^ = 0.9843). GdNP loaded SRBs in tumor were clearly visible up to at least 14 days post-implantation. Signal decrease during this time showed GdNP release in vivo, which was calculated as 3.86 ± 0.34 µg GdNPs release into the tumor. This study demonstrates potential and feasibility for SRBs with MRI-contrast, and sensitive GdNP quantification and release from SRBs in a preclinical animal model. The feasibility of monitoring nanoparticle (NP) concentration during treatment, allowing dynamic quantitative treatment planning, is also discussed.

## 1. Introduction

Cancer is one of the leading causes of death worldwide [1] and more than 50% of cancer patients receive radiation therapy (RT) either as a standalone treatment or in combination with other treatment modalities like chemotherapy, surgery or immunotherapy [2]. During RT the goal is to kill cancerous cells by delivering a physician-prescribed dose of radiation to the target tumor; at the same time damage to the surrounding normal tissue should be minimized. Technological advances in radiation oncology allow for increased sparing of healthy tissue while maintaining or even improving tumor coverage. These advances often profit from implantable RT materials such as fiducials or beacons for providing imaging contrast. These RT materials are employed to ensure geometric accuracy and tracking of tumors that move during treatment, e.g., due to respiratory motion. The potential of upgrading these RT materials to fulfill a dual purpose of providing imaging contrast and radio-enhancing properties has been proposed [3]. Here we demonstrate how currently used RT materials can be replaced by smart radiotherapy biomaterials (SRBs) [3,4], that can be loaded with nanoparticles (NPs) [5] to provide magnetic resonance imaging (MRI) contrast. The use of implants to deliver certain payload in cancer treatment is an ongoing research topic and includes the use of different polymers [6,7] and different designs, which have taken the form of gels, nanoparticles, polymeric films, rods, and wafers [8]. Such localized therapies of a drug controllably delivered into the disease site can significantly reduce tissue toxicities [9,10]. With respect to radiation oncology, the payload of high-atomic number NPs in the SRBs has been suggested [3,4,11] and studied in silico [12], in vitro [13], and in vivo [5]. Under irradiation, such NPs can exhibit increased radiation dose in their close proximity due to physical interaction of the ionizing radiation with the high-atomic number material of the NP. At the same time, these NPs can provide imaging contrast, as currently achieved in RT by fiducials or beacons. Hence, these inert materials used for geometric accuracy could be replaced by SRBs, which additionally provide radiation dose enhancing properties by releasing the NPs into the tumor, which can include drug payloads such as immunoadjuvants [14]. In the present study, the imaging capability of a loaded SRB is investigated and the payload release is modelled for accurate prediction. The potential dose enhancement under irradiation is discussed.

In considering NPs for loading SRBs, gadolinium-based NPs (GdNPs) can provide imaging contrast during MRI. Gadolinium is commonly used during MRI because of its seven unpaired electrons, making it one of the most paramagnetic stable metal ions [15]. The spin-lattice relaxation time (T_1_) will be shortened for voxels in which Gd is present and will consequently appear brighter in T_1_-weighted images [15]. The GdNPs used for this study, AGuIX^®^, have been developed for imaging due to their MR contrast properties but have also been shown to improve radiotherapeutic efficacy [16] and are currently in clinical trials [17]. For favorable biodistribution these GdNPs are manufactured ultrasmall. In addition, these NPs showed low toxicity to cells and are well tolerated in absence of radiation [16,18].

In this study, these GdNPs are loaded into an SRB. Such NP-loaded SRBs will hence present imaging contrast similar to routinely implanted fiducials/beacons. For such an application, it is essential to (1) demonstrate the ability of providing imaging contrast over the time of radiation treatment and (2) to quantify the release of GdNPs. This study investigates the ability of SRBs loaded with GdNPs to provide MR imaging contrast in vivo and quantifies the release of GdNPs into the tumor with the potential to increase the dose gradient due to radiosensitization and dose-painting with NPs. Potential application due to radiosensitization by the NPs, in addition to providing imaging contrast, as well as limitations of radiation necrosis on the T_1_ signal will be discussed.

## 2. Materials and Methods

### 2.1. Gadolinium-Based Nanoparticles (GdNPs)

**Figure 1:**
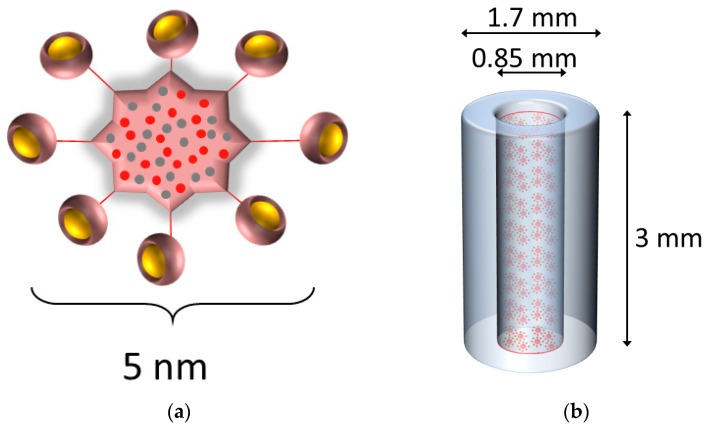
Gadolinium-based NPs loaded into an SRB. (**a**) Schematic representation of the GdNPs. Gadolinium ions chelated to DOTA ligands (yellow) grafted on a polysiloxane matrix comprising mainly silicon and carbon (red and grey). The overall size as hydrodynamic diameter is about 5 nm. (**b**) The gadolinium-based NPs (not to scale to SRB) were dispersed in deionized water and loaded into the SRB. The SRB (blue) is 3 mm in length and 1.7 and 0.85 mm in outer and inner diameter, respectively. Gd = gadolinium, NP = nanoparticles, SRB = smart radiotherapy biomaterial.

Freeze-dried spherical 5 nm sized (hydrodynamic diameter) gadolinium-based nanoparticles, AGuIX^®^, were provided by NH Theraguix (Lyon, France). They consist of Gd ions surrounded by DOTA chelators, of which eight are attached to one polysiloxane shell as shown in Figure 1a. One mg of GdNPs contains about 1 µmol of Gd [19]. AGuIX^®^ synthesis and characteristics have been described previously [20,21]. GdNPs were dispersed in deionized water and sonicated for 10 min for homogenization prior to being used. Dispersion of freeze-dried NPs to NPs in solution results in no noticeable hydrodynamic diameter change (less than 5% difference) and stability of colloidal GdNP suspension has been confirmed [20].

### 2.2. Agar Samples

0.2% agar samples were created by stirring noble agar (Sigma-Aldrich, St. Louis, MO, USA) into boiling de-ionized water until completely dissolved, pouring into a glass container (20 mL volume) and curing before closing the container. Into these agar samples GdNP solution was injected using a 10 µL pipette.

### 2.3. Smart Radiotherapy Biomaterial (SRB)

SRBs were fabricated using a mixture of poly(lactic-co-glycolic) acid (PLGA) polymer (Aldrich, St. Louis, MO, USA) and acetone (Sigma, St. Louis, MO, USA) following previously established protocols [5,14]. In brief, the PLGA-acetone mixture is loaded into a silicon tubing using the Harvard apparatus, dried for 72 h at 50 °C, and then cut to sizes like currently used fiducial markers. The resulting SRB has the shape of a hollow cylinder and is 3 mm in length and 1.7 mm in outer diameter and 0.85 mm in inner diameter. The hollow core of the SRB was filled with 1.2 µL of a 4.1 mg/mL GdNP concentration in deionized water (Figure 1b). The ends of the SRB are sealed using the same PLGA-acetone mixture and air dried. One SRB was injected per tumor using clinical brachytherapy needles holding a mass of 4.92 µg GdNPs, corresponding to 0.246 mg GdNP/kg body weight. Comparison to SRB delivery by direct intratumoral injection of GdNPs was performed by injecting 20 µL of the 4.1 mg/mL GdNP concentration using a needle.

### 2.4. Mouse Tumor Model

**Figure 2:**
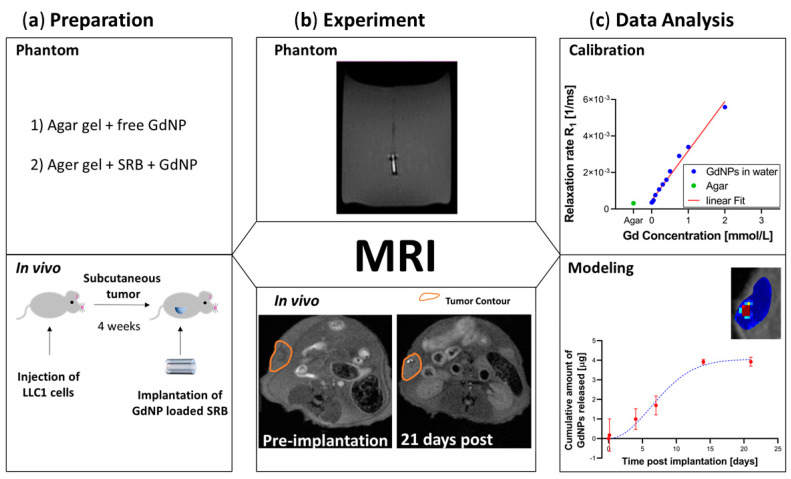
Overview of the study design. (**a**) Preparation. Agar gel was utilized as the phantom material prior to in vivo testing in animals. (**b**) Experiment. MRI was utilized on phantoms and in vivo to achieve T_1_-weighted images and consequently T_1_-maps for quantification over time. (**c**) Data Analysis. Calibration of relaxation rate R_1_ allows for modeling the release profile of GdNPs from SRB. MRI = magnetic resonance imaging, GdNPs = gadolinium nanoparticles, SRB = smart radiotherapy biomaterial.

Animal experiments were conducted compliant with guidelines and regulations set by the Institutional Animal Care and Use Committee (IACUC) of the Dana-Farber Cancer Institute (protocol 15-040 approved on 5 January 2016 and protocol 08-023 approved on 1 November 2019). Lewis Lung carcinoma cells LL/2 (LLC1) were obtained from American Type Culture Collection (ATCC, Manassas, VA, USA) and were cultured in Dulbecco’s Modified Eagle Medium (DMEM, Thermo Fisher Scientific, Waltham, MA, USA), supplemented with 1% penicillin/streptomycin (Thermo Fisher Scientific, Waltham, MA, USA) and 10% fetal bovine serum (Thermo Fisher Scientific, Waltham, MA, USA). Immunocompetent wild-type C57BL/6 male mice (Taconic Bioscience, Rensselaer, NY, USA) were subcutaneously inoculated with 50,000–100,000 live LLC1 cells, as determined by trypan blue staining (Lonza, Basel, Switzerland), for tumor generation. Tumor growth was monitored and around 4 weeks after cell inoculation the tumors were considered mature for MR imaging (Figure 2a).

### 2.5. MR Imaging

Vials containing known concentrations were used for calibrating relaxation rates (R_1_) to Gd concentration. Gd concentration measurements were subsequently performed for free GdNPs and loaded into an SRB in vivo in an animal model for investigation.

MRI experiments were performed on a 7T Bruker BioSpec superconducting magnet system (Bruker Corp, Billerica, MA, USA) with a 30 cm USR horizontal bore. The system provides a maximum gradient amplitude of 440 mT/m and slew rate of 3440 T/m/s. The Bruker made 35 mm diameter birdcage volume radiofrequency (RF) coil was used for RF excitation and receiving. The Bruker AutoPac positioning was employed for accurate region of interest positioning. The Bruker Paravision 6.0.1 was used for MRI data acquisition.

The phantom study included MRI acquisitions of deionized water loaded at several GdNP concentrations; this was used for calibration. Free GdNP and SRB + GdNP were studied in agar to mimic the subsequent in vivo study (Figure 2a,b).

For the in vivo part, mice were placed on a stereotactic frame and anesthetized for the duration of the procedure through inhalation of a mixture of 1.5% isoflurane and oxygen. The anesthetic was maintained at a flow rate of 2 L/min. Body temperature was set at 37 °C using a warm air fan. Mice temperature and respiration were monitored using a pressure-transducer placed on the abdomen for respiratory gating. Mice were imaged prior to injection of the SRB or free GdNPs and at several time points post injection (Figure 2b). Four subjects with GdNP-loaded SRBs were investigated, and the imaging time points were 10 min, 3 h or 1 day, 4 days, 7 days, 14 days, and 21 days post-implantation. The control of free GdNPs included one subject, with imaging time points at 10 min, 3 h, 8 h, 1 day, 4 days, and 7 days post-injection. The control of GdNP-free SRB was tested in one subject, measured at 10 min, 1 day, and 7 days post-injection. T_1_-weighted images were adjusted in brightness to allow direct comparison.

A fast spin echo (FSE) sequence was used for both phantom and in vivo experiments to generate T_1_-weighted images for positioning purposes, as well as for T_1_ maps. The T_1_-weighted sequence parameters were: echo time (TE) = 16 ms, repetition time (TR) = 553 ms, number of averages (NSA) = 2, echo train length (ETL) = 2, matrix size = 128 × 128 × 3, field-of-view = 0.3 × 0.3 mm^2^, thickness = 1 mm, and scan time = 35 s. The scanning parameters for the T_1_ map sequence were as follow: 10 T_1_ experiments corresponding to TR = 7000, 5000, 4100, 3400, 2500, 1800, 1300, 800, 400, and 278 ms, matrix size = 128 × 128 × 3, field-of-view = 0.23 × 0.23 mm^2^, thickness = 1 mm, and scan time = 21 min 20 s. The T_1_ map unreported parameters were similar to the T_1_-weighted sequence. T_1_ maps were generated by an internal application of Bruker Paravision, where the 10 T_1_ experiments were fitted on a pixel base to obtain pixel-by-pixel T_1_ values. The T_1_ maps were then saved and exported offline for further MATLAB^®^ analysis.

### 2.6. MR Calibration

For studying Gd release, the MRI system was calibrated using known concentrations of Gd. Gd increases both the longitudinal (R_1_ = 1/T_1_) and transverse (R_2_ = 1/T_2_) relaxation rates of the solvent nuclei. The observed relaxation rate $1/T_{i,obs}$ (where *i* = 1,2 for longitudinal and transverse relaxation, respectively) is the sum of diamagnetic $1/T_{i,dia}$ and paramagnetic $1/T_{i,para}$ relaxation rates [15]. Whereas the diamagnetic relaxation rate corresponds to the solvent in absence of the solute, the paramagnetic relaxation rate originates from the dipole–dipole interaction of nuclear spin and the magnetic field fluctuating as cause of the unpaired electron spin of the paramagnetic substance. The paramagnetic relaxation rate is linearly proportional to the concentration of the paramagnetic substance, here the substance is Gd. The proportionality constant is the relaxivity $r_{i}$ [15].

(1) $$\begin{array}{c} \frac{1}{T_{i,obs}}=\frac{1}{T_{i,dia}}+\frac{1}{T_{i,para}}=\frac{1}{T_{i,dia}}+r_{i}\cdot \left[\mathrm{Gd}\right],\\ \mathrm{for}\;i=1,2\;\mathrm{for}\;\mathrm{longitudinal}\;\mathrm{and}\;\mathrm{transverse}\;\mathrm{relaxation},\;\mathrm{respectively}. \end{array}$$

Equation (1) models a linear relationship for observed relaxation rate versus paramagnetic substance concentration, where the slope corresponds to the relaxivity $r_{i}$. MR calibration was performed according to previously described methods [15], which have been successfully used for application of NPs [22,23]. Vials of known Gd concentrations between 0 and 2 mmol/L were scanned and T_1_ maps generated. From these relaxation times T_1_ corresponding relaxation rates R_1_ were calculated and used for calibration (Figure 2c). Following Equation (1) the relaxivity *r_1_* can be used for calculating the Gd concentration for a given T_1_, as previously demonstrated for AGuIX^®^ NPs [24].

### 2.7. Release Kinetics

Determining the Gd concentration using this method allows determining the amount of Gd left in the SRB and concomitantly the mass that has been released from the SRB. The release kinetics of this mass transport are in good agreement with the Weibull model [25]. Compared to other release models, it covers several release variants, as pure Fickian diffusion or anomalous transfer [26]. Hence, the Weibull model can be used to indicate a drug release mechanism and is expressed as:

(2) $$\frac{M_{t}}{M_{\infty }}=1-\exp\left(-a\cdot t^{b}\right),$$

where $M_{t}$ and $M_{\infty }$ are the mass that is released at time *t* resp. infinity, *a* is a scale parameter and *b* is a shape parameter characterizing the shape of the curve and is hence characteristic for the release mechanism [26] (Figure 2c).

## 3. Results

**Figure 3:**
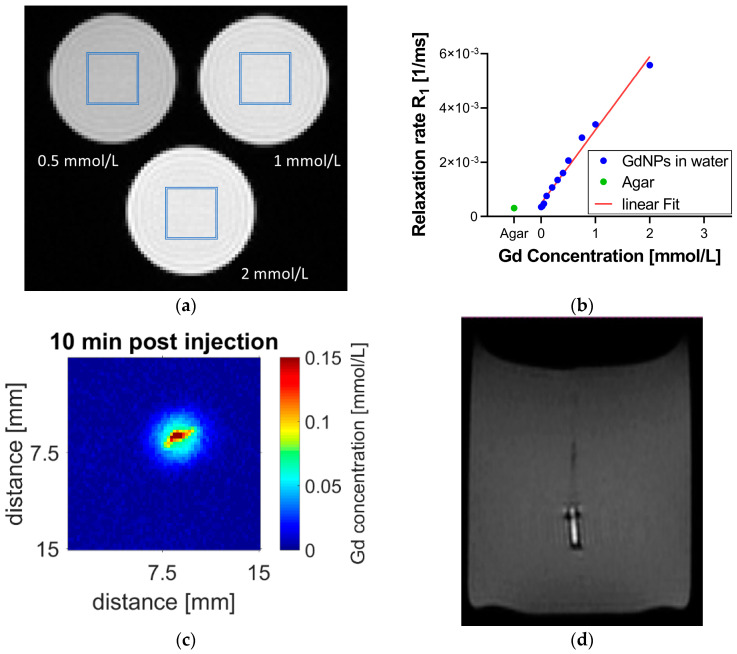
MR calibration and SRB visibility after implantation into agar gel. (**a**) Vials of known Gd concentration were used for MR calibration. The regions of interest in the center of the vial are highlighted (blue). (**b**) Linear fit of R_1_ relaxation rate versus Gd concentration (blue) with a detection threshold of 0.004 mmol/L. This calibration can be used to assign Gd concentration to measured T_1_ data on a pixel-by-pixel basis. This is demonstrated in (**c**) depicting an injection of GdNPs into an agar phantom sample. The agar sample itself has no Gd; Gd concentrations are measured only around the injection site. (**d**) The GdNP-loaded SRB shows good contrast compared to agar. The bright signal originates from the GdNPs in its center while the SRB sides report no MR signal and thus appear dark, similar to air. MR = magnetic resonance, SRB = smart radiotherapy biomaterial, Gd = gadolinium, NP = nanoparticle.

To determine the relaxivity *r_1_*, relaxation time T_1_ was measured for several known concentrations of GdNPs dissolved in deionized water. Figure 3a shows the T_1_-weighted image of 3 vials of Gd concentrations (0.5, 1, and 2 mmol/L) imaged at once. From the corresponding T_1_ map, relaxation rates for each concentration are determined. Figure 3b displays R_1_ relaxation rates of the 10 vials examined, which were linearly fitted to Gd concentration with a high confidence level (R^2^ = 0.9843), revealing a relaxivity of *r_1_* = 2.7 mM^−1^ s^−1^ and a detection threshold of 0.004 mmol/L as determined following standard literature [27]. Figure 3b further shows the R_1_ relaxation rate measured for the agar sample (green) being in good agreement with the relaxation rate between that of the agar sample and that of deionized water in absence of Gd. Figure 3c displays the distribution of free GdNPs in an agar gel sample. The Gd concentration map was created pixel-wise (by an in-house MATLAB^®^ script) by combining T_1_ and the Gd calibration map. Figure 3d reports a T_1_-weighted MR image of the GdNP-loaded SRB in agar. The GdNPs in the SRB center provide a strong signal; the SRB itself, on the contrary, appears much darker, similar to air, most probably due to the lack of free protons in its molecular matrix.

**Figure 4:**
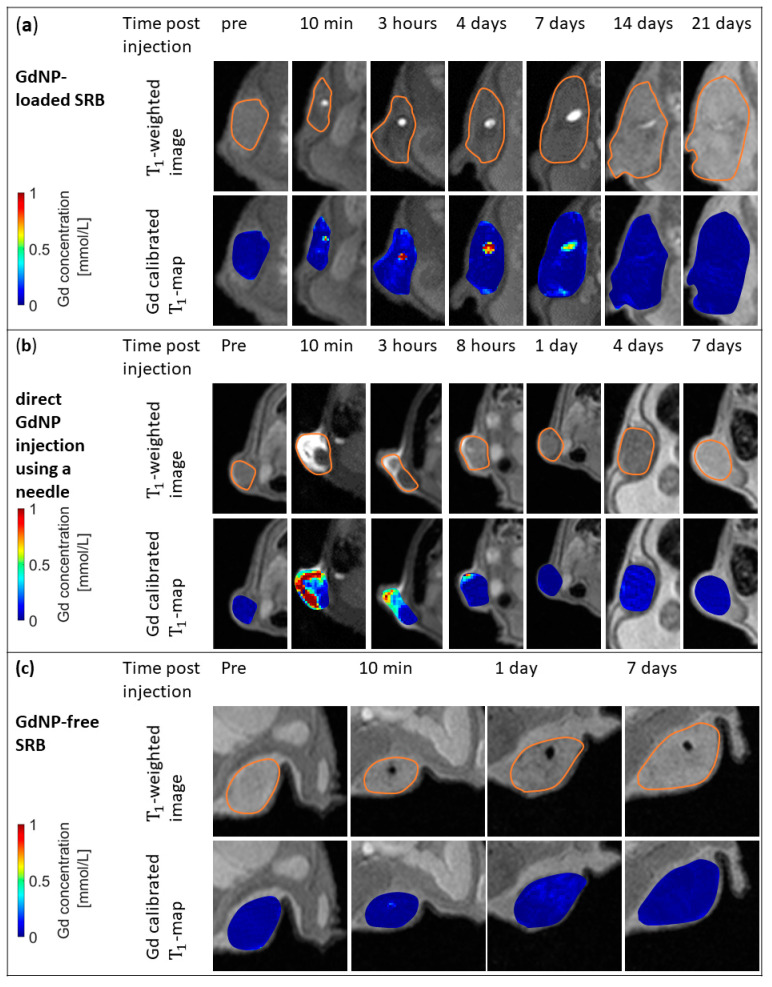
In vivo MR images and maps for GdNP-loaded SRB, direct GdNP injection, and GdNP-free SRB. (**a**) T_1_-weighted MRI (top row) and Gd-calibrated T_1_ maps of the subcutaneous tumor overlaid onto T_1_-weighted images (bottom row) of a mouse prior to and at several time points post-injection of a GdNP-loaded SRB. The loaded biomaterial is clearly visible inside the tumor and descending concentrations of Gd within the biomaterial are observed. (**b**) Images show the direct injection of GdNPs into the tumor using a needle. Signal at 10 min post-injection derived by free GdNPs vanishes after 1 day. (**c**) GdNP-free SRB indicates that T_1_-signal is originating from GdNPs only. SRB = smart radiotherapy biomaterial, MRI = magnetic resonance imaging, GdNPs = gadolinium-based nanoparticles.

In addition to the T_1_-weighted images, T_1_-maps were generated, allowing the quantification of Gd concentration in SRB. A sustained image reference up to at least 14 days post SRB implantation is observed, while the fading of the SRB is noticeable. Figure 4a shows the good alignment between the bright signal in the T_1_-weighted images and Gd concentrations according to the T_1_-map data inside the tumor. The sustained image reference of at least up to 2 weeks is only provided by loading the GdNPs into an SRB. When administering the GdNPs as intratumoral injection (Figure 4b) the signal caused by the GdNPs vanishes within 1 day. Confirmation of the signal originating from GdNPs can be seen in Figure 4c, in which a GdNP-free SRB was implanted, but does not show the T_1_-signal.

**Figure 5:**
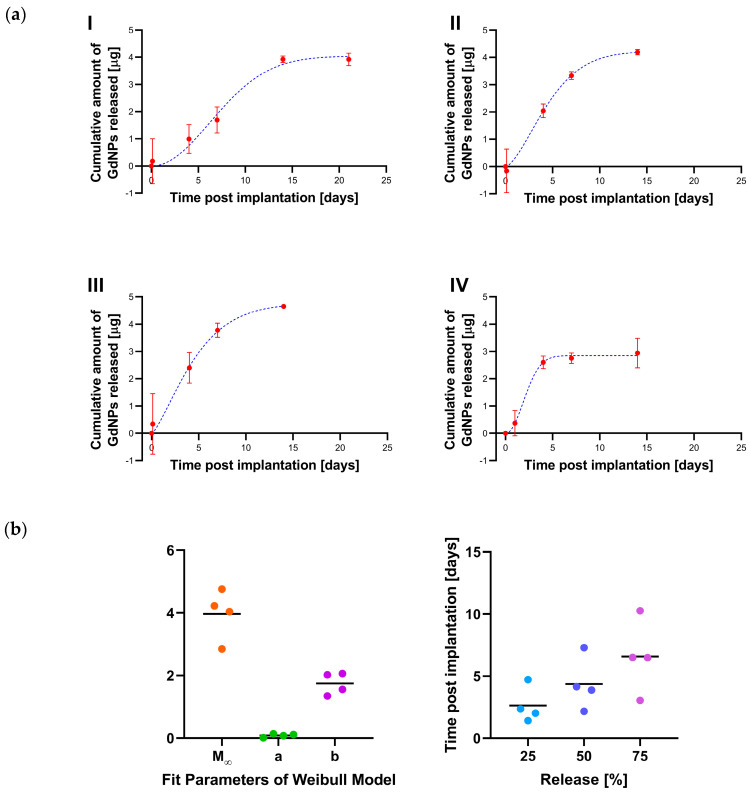
GdNP release from SRB. (**a**) The amount of GdNP released from the SRB as a function of time is shown for individual mice (red). The Weibull function is fitted to these data (blue) and (**b**) Fitting parameters of the Weibull distribution allow prediction of release. SRB = smart radiotherapy biomaterial, GdNPs = gadolinium-based nanoparticles.

The GdNP release from the SRB is quantitatively described in Figure 5a. From the Gd concentration inside the SRB as a function of time, the GdNP release into the tumor is calculated for each individual mouse. The Weibull function is fitted to the cumulative released mass, indicating a total of 3.86 ± 0.34 µg GdNPs were released on average from the SRB within 14 days. The shape factor *b* of the Weibull function is determined as 1.75 ± 0.15, ranging between 1.35 and 2.06. Hence, for each individual mouse the shape factor was larger than 1. This sigmoid shape is in accordance with a release mechanism not described by Fickian diffusion, but rather with a release rate first increasing up to an inflection point and then declining [26]. The Weibull model further allows prediction of release with on average 2.6, 4.4, 6.6, and 13.5 days until 25%, 50%, 75%, and 99% of GdNPs are released, respectively (Figure 5b).

Overall, the results indicate that SRBs loaded with GdNPs can provide image contrast and hence could be used similar to currently used inert radiotherapy biomaterials like fiducials. Furthermore, the study shows sustained release and retention of the GdNPs in the tumor over many days compared to the direct intratumoral administration. The analysis indicates the cumulative release equals between 5 days and 15 days. The Weibull distribution indicates a shape parameter *b* > 1, consistent with non Fickian diffusion of GdNPs from the SRB. This finding is valuable towards development of SRBs for multi-functional applications in radiation oncology.

## 4. Discussion

In this study quantitative concentration measurements of GdNPs loaded into next generation SRBs were performed in an in vivo animal model. To this end, vials of known concentrations of Gd were used to calibrate relaxation rates R_1_ to Gd concentrations with a detection threshold of 0.004 mmol/L. The calibration allowed for determining Gd concentrations in different tissues [28,29] as shown in this study for free GdNPs in an agar sample and for SRB loaded with GdNPs in an animal model. In vivo, the GdNP loaded SRB is clearly visible using MRI for at least 14 days post-implantation. The SRB degradation process was observed as NPs were released and this release of NPs into the tumor tissue was quantified. Analysis using the Weibull function indicates that the release kinetics might not be a sole diffusion process, but rather that the release rate varies with time, reporting a rapid increase in the first few days, followed by a reducing rate. Using this model, it was further calculated that it takes on average 4.4 days to release 50% of the GdNPs from the SRB. Over a 14-day time period, 3.86 ± 0.34 µg GdNPs are released on average.

Gd for in vivo applications must be chelated to organic ligands to overcome toxicities in humans. The GdNPs used in this study utilize DOTA chelators approved by the Agence Nationale de Sécurité du Médicament et des produits de santé (ANSM), and their safety, tolerability, and side-effects were studied in a first in man phase Ib clinical trial (NANORAD, NCT02820454) [17,19]. The dose of GdNPs used in the presented study is two orders of magnitude lower than under clinical investigation and less than 0.01% of the maximum tolerated dose that has been previously determined for mice after intravenous injection [30]. It is expected that tolerance and excretion routes and times will depend on the route of administration. However, the currently available toxicity data support safe application of these GdNPs in vivo. It is further expected that in situ delivery approaches as by using SRBs will minimize systemic toxicities [9,10].

The field strength of the magnetic field utilized in this study (7T) was twice as strong as MR-Linacs which operate at a field strength below 3.5T and consequently the *r_1_* values are expected to be lower, resulting in a stronger signal than expected from the MR-Linac [31,32]. In our study, due to the use of small molecules, a change in *r_1_* values might be less pronounced than for larger molecules, e.g., caused by surface coatings [23,32]. Relaxivity *r_1_* at 7T equals 2.7 mM^−1^ s^−1^ per Gd^3+^ ion, resulting in a relaxivity *r_1_* per GdNP of 21.6 mM^−1^ s^−1^. Image quality of T_1_ map data are further limited by geometric constraints, motion artifacts or increased field inhomogeneities in proximity of the RF coil. Our data, nonetheless, reported minimal motion artifacts and an absence of field inhomogeneities artifacts, confirming good image quality, which in turn was reflected in an apparently accurate T_1_ map analysis. Image ringing artifacts (known as Gibbs artifacts), which commonly occur at low image resolution, are observed in the phantom study (Figure 3a) [33]. Such effects, although contributing to uncertainties of the calibration (Figure 3b), were minimized by placing the region of interest for data analysis calibration in the center of the vials so as to avoid the strongest effects experienced in the outer part. In addition, relaxivity measurements are temperature dependent [34,35], a factor which is currently not considered. More experimental studies will be needed to evaluate the impacts of field strength and temperature dependence.

The repeat study showed reproducibility of the acquired data and it is worth mentioning that for every mouse out of four the Weibull distribution indicated a shape parameter *b* > 1, consistent with non Fickian diffusion of GdNPs from the SRB. However, more studies are required to establish reproducibility with varying NP concentration and SRB size, as well as drug-loading with immunoadjuvants. Such immunoadjuvant therapies have the potential to contribute to improving cancer treatment by transforming the local effects of radiation therapy towards a systemic response, attacking cancerous cells outside the irradiated field. Delivery of such immune-stimulatory agents by NPs [36], or sustained delivery by SRBs [3,4] and sodium alginate injections [37] are currently under investigation. The work presented in this study is valuable in guiding diverse investigations of new radiotherapy biomaterials for providing sustained image contrast in radiation oncology, with the potential for release of the payloads to enhance local therapy outcomes.

The model used to describe GdNP release conforms with a non-monotonic release of NPs from the SRB in vivo with an increasing release rate followed by a decrease. This would be consistent with previously reported NP/drug loaded biomaterials, for which a burst release was observed initially before the release slowed down [5,38]. Nagesha et al. suggested loading another layer to prevent this burst release [38], making these biomaterials tunable to serve different purposes [13]. The use of an MRI-based calibration, as demonstrated in the present study, will allow comparing different SRB designs and creating tailored SRBs with tunable qualities such as capacity, release rate, and imaging contrast. Previous theoretical studies used simplistic models of Fickian distribution for a first estimate on gold NP eluters in combination with brachytherapy sources [12]. Findings presented in this study suggest that release might not follow diffusion-based release mechanisms; however, findings showing an advantage of using brachytherapy sources with longer half-lives are expected to hold even for nonlinear release. Tuned AGuIX^®^ NPs with additions of different metals could further provide different modalities to their MR contrast. Europium and Terbium offer fluorescent properties [19] and radioactive isotopes can be utilized for PET/SPECT imaging and brachytherapy [19,39].

The use of SRBs has been proposed; they are intended to replace currently used RT materials and fulfill dual purposes [3]: on the one hand, they provide imaging reference during RT, and on the other hand they deliver NPs as their payload directly into the tumor, which will act under irradiation as a radiosensitizer. This study demonstrates the delivery of GdNPs into the tumor and the sustained image reference provided by the GdNP-loaded SRB over at least 14 days in an in vivo animal model exposed to bodily processes and microenvironments. As such, the GdNP-loaded SRB provides a signal over a longer time than that after direct intratumoral injection or after intravenous injection, as demonstrated for glioma-bearing rats [40]. For AGuIX^®^ the radiosensitization has been demonstrated for different radiation sources and energies in various in vitro and in vivo animal models, as reviewed by Sancey et al. [16]. Dose enhancement factors caused by AGuIX^®^ under clinical 6 MV radiation has been shown to be of the same magnitude as for gold NPs under similar experimental conditions [41]. Based on this known radiosensitization for AGuIX^®^ NPs, further studies can elaborate on the radiotherapeutic outcome of the radiosensitizing GdNP release from SRB in comparison to different routes of administration.

## 5. Summary and Outlook

This study successfully demonstrated that SRBs loaded with GdNPs provide an image reference during MRI over at least 14 days. During this time, Gd concentration can be quantified, allowing for calculation of released NP mass. These NPs are released into the surrounding tumor, where they may lead to enhanced tumor cell kill during irradiation [41,42]. This represents the basis for quantitative RT planning using NPs with the potential for use in MR-Linacs, which can monitor the NP concentration. However, careful investigation will be required to evaluate if T_1_ signal change is originating from the GdNPs. Such T_1_ signal change can be caused by radiation necrosis. Radiation necrosis is a late outcome of irradiation that occurs several months after treatment [43], such that it is expected to be negligible at the beginning of RT, when GdNP concentrations are highest. This impact must be studied when adding radiation. The application of utilizing the T_1_ signal for Gd concentration measurements in the absence of radiation was demonstrated in this study. Such real-time information is the foundation for a dynamic treatment planning scenario. The results demonstrate sustained image-guidance and delivery of GdNPs using SRBs in a preclinical animal model. This is an important step towards RT, with potential for use in MR-Linacs and dose-painting with NPs.
